# Mechanical Properties and Metal-Ceramic Bond Strength of Co-Cr Alloy Manufactured by Selective Laser Melting

**DOI:** 10.3390/ma13245745

**Published:** 2020-12-16

**Authors:** Joon-Ki Hong, Seong-Kyun Kim, Seong-Joo Heo, Jai-Young Koak

**Affiliations:** Department of Prosthodontics and Dental Research Institute, Seoul National University Dental Hospital, School of Dentistry, Seoul National University, 101 Daehak-ro, Jongno-gu, Seoul 03080, Korea; hadley@snu.ac.kr (J.-K.H.); heosj@snu.ac.kr (S.-J.H.); young21c@snu.ac.kr (J.-Y.K.)

**Keywords:** Co-Cr alloy, 3D printing, selective laser melting, Metal-Ceramic bond strength, mechanical properties

## Abstract

Cobalt–chromium (Co-Cr) metal is one of the widely used biomaterials in the fabrication of dental prosthesis. The purpose of this study was to investigate whether there are differences in the properties of metals and bond strength with ceramics depending on the manufacturing methods of Co-Cr alloy. Co-Cr alloy specimens were prepared in three different ways: casting, milling, and selective laser melting (SLM). The mechanical properties (elastic modulus, yield strength, and flexural strength) of the alloys were investigated by flexure method in three-point bending mode, and microstructures of the specimens were analyzed. After application of the veneering ceramic through the three-point bending test, bond strength of the Metal-Ceramic was investigated. The cracked surfaces were observed by means of energy dispersive X-ray (EDX) spectroscopy and scanning electron microscopy (SEM) with backscattered electron (BSE) images. In mechanical properties, the elastic modulus was highest for the casting group, and the yield strength and flexural strength were lowest for the milling group. The SLM group showed finer homogeneous crystalline-microstructure, and a layered structure was observed at the fractured surface. After the ceramic bond strength test, all groups showed a mixed failure pattern. The casting group showed the highest bond strengths, whereas there was no significant difference between the other two groups. However, all groups have met the standard of bond strength according to international standards organization (ISO) with the appropriate passing rate. The results of this study indicate that the SLM manufacturing method may have the potential to replace traditional techniques for fabricating dental prosthesis.

## 1. Introduction

With the development of computer-aided design/computer-aided manufacturing (CAD/CAM) technology, CAD/CAM prostheses are often used these days in dentistry [1]. In recent decades, Metal-Ceramic prostheses made of cobalt–chrome (Co-Cr) alloys have been used extensively [2]. Co-Cr alloy is one of the most widely used dental alloys due to its low price, good corrosion resistance, and high mechanical strength [3].

The casting method, most commonly used when producing Co-Cr frameworks, is applied by producing and casting a wax pattern. This conventional method is labor-intensive, can cause errors in the manufacturing process, and is prone to human mistake [4]. Fortunately, recent developments in CAD/CAM technology have made it possible to create Co-Cr frameworks by means of nontraditional methods [5].

Two of the new methods are milling and selective laser melting (SLM). It is expensive to purchase new software and equipment, and it takes time to learn new skills and knowledge to use the new CAD/CAM manufacturing processes well, as they are different from the traditional way [6,7]. However, the CAD/CAM manufacturing methods have more advantages. These are less time-consuming and more suited to mass production than the casting method. Co-Cr is an alloy that is often produced via CAD/CAM fabrication methods and is also frequently used in producing porcelain fused to metal (PFM) restorations [8]. In addition, Co-Cr alloys are used more often than Ni–Cr alloys because of their superior biocompatibility [9,10].

The milling technique is a subtractive method whereby a metal structure is formed by cutting a prefabricated metal disk. Whereas casting can produce defects or dimples in the cast structures, milling can avoid this problem because the disc used can be manufactured in a highly standardized industrial environment [11]. The SLM technique is an additive method in which a structure is built up from fine layers of metal powder by melting the areas to include with a high-power laser beam. The SLM manufacturing method has several advantages. Complex structures can be produced with minimal material waste, human error by technicians is reduced, and better quality products can be made due to improved productivity. Moreover, production costs can be reduced through mass production. In addition, because SLM takes less manufacturing time, it can provide greater benefits to patients and clinicians [12].

Before clinically using a material produced in a new way, it is necessary to ensure that its various properties are clinically appropriate. Such properties include biocompatibility, corrosion resistance, marginal fit, and mechanical properties. Although there are various relevant mechanical properties, the yield strength and flexural strength are the most important factors because the metal should not fracture or deform under masticatory forces. The most common clinical complication in the use of Metal-Ceramic prostheses is fracture of the ceramic area [13]. Therefore, the Metal-Ceramic bond strength is also important [14].

Despite the many advantages of the SLM method, little research has been conducted on the properties of the metals produced and their bond strength with ceramics. Among the various test methods, the three-point bending test is widely used as the standard for measuring Metal-Ceramic bond strength and is also listed in the ISO regulations for dental restorations [15,16].

The objective of this study was to examine how Co-Cr metals fabricated by means of various methods differed in Metal-Ceramic bond strength and mechanical properties.

## 2. Materials and Methods 

### 2.1. Preparation of Metal Specimens for Mechanical Properties Testing

**Figure 1:**
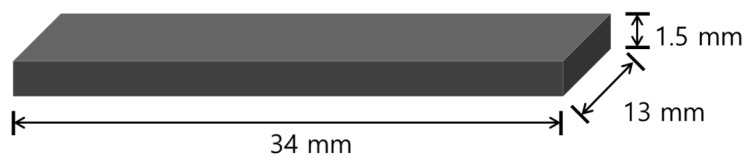
Schematic diagram of the shape of the alloy specimens.

Metal specimens for mechanical properties testing (Figure 1) were manufactured by means of milling, casting, and SLM (*n* = 12 each group). For the casting group Co-Cr cast specimens were made by means of conventional lost-wax casting. Wax was made into a plate shape as a template and mounted in a casting ring. Samples were invested in a phosphate-bonded investment (Bc-vest Cb-formula, Bukwang, Seoul, Korea). The rings were later put into a furnace (Miditherm 100MP, BEGO, Bremen, Germany) to evaporate the wax. The casting was made using Co-Cr alloy ingots (Star Loy C, Dentsply Sirona, York, PA, USA) in a casting device (Casting machine, Seki Dental, Seoul, Korea) according to the manufacturer’s instructions. The casting was allowed to cool to room temperature and no post-production heat treatment was applied.

CAD software (3shape CAD, 3shape, Copenhagen, Denmark) was used to design the milling group specimens and the data were transferred to CAM software (HyperDENT, 3DBioCAD, Washington, DC, USA). Specimens were made by milling from a premanufactured Co-Cr alloy disk (Starbond Co-Cr block, Scheftner Dental Alloys, Mainz, Germany) using a milling machine (Arum 5x-200, Arum, Frankfurt, Germany). The milling procedure was followed by the manufacturer’s instructions.

The SLM group specimens were designed by using CAD software (EOS RP Tools, EOS, Krailling, Germany) and transferred to CAM software (3shape Cambridge, 3shape). Subsequently, specimens were printed using a 3D printer (EOSINT m270, EOS) equipped with a 200 W Yb-fiber laser, using Co-Cr powder (SP2, EOS) and building vertically. The manufacturing parameters were selected according to the manufacturer’s instructions. The size of the Co-Cr powder was 10–45 µm, the building layer thickness was 20 µm and the scan speed used during building was up to 7.0 m/s. Post-production heat treatment was applied in a muffle furnace (Muffle furnace 1000, Daeheung, Incheon, Korea) at 700 °C for 50 min.

After all specimens were fabricated, unnecessary parts of their metal surfaces were removed using stone points, and the surfaces were polished using rubber points. Afterward, the samples were sandblasted with 80 µm Al_2_O_3_ particles under 4 bar pressure for 5 s, and then cleaned with an ultrasonic cleaner. Table 1 lists the compositions of the alloys used to fabricate the specimens.

### 2.2. Surface Characterization

For surface characterization, five random specimens were selected per fabrication group, and the average surface roughness (*R_a_*) of the base metal was determined using a confocal laser microscope (Zeiss LSM 800 MAT & Zeiss Axio imager Z2m, Zeiss, Jena, Germany) equipped with ZEN software (Zeiss). Imaging was performed using laser excitation at 405 nm with a 20×, 0.7 numerical aperture lens over evaluation lengths of 319 µm. The tests were performed at three different points on each specimen. 

### 2.3. Mechanical Properties Testing and Microstructure Analysis

**Figure 2:**
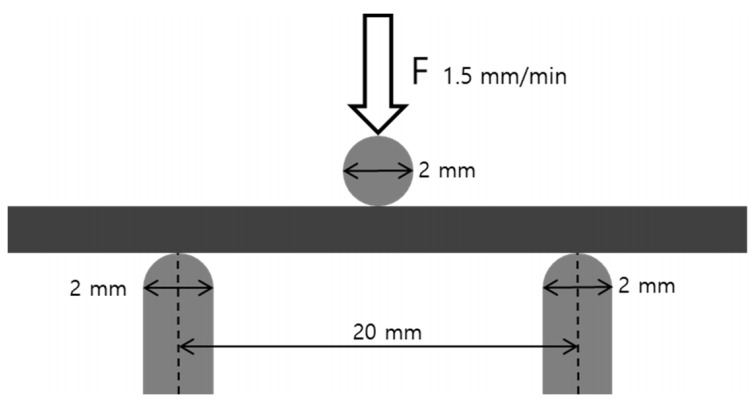
Schematic diagram illustrating the three-point bending test used to measure mechanical properties. The distance between the supports was 20 mm and the diameters of the bending piston and supports were each 2 mm. The bending piston was placed at the center and the crosshead speed of the loading part was 1.5 mm/min.

Three-point bending tests were performed on the metal specimens according to ISO 22674:2016 using a universal testing machine (Instron 8871, Instron, Norwood, MA, USA); the crosshead speed of 1.5 mm/min was applied until the specimen fractured. The distance between the supports was 20 mm and the diameter of the bending piston was 2 mm (Figure 2).

The 0.2% yield strength and flexural strength were calculated from the recorded load and crosshead movement. Flexural stress and flexural strain were computed as

$${\mathrm{Flexural}\;\mathrm{stress}\;\sigma }_{f}=\frac{3\mathrm{FL}}{2{\mathrm{bd}}^{2}}$$

$${\mathrm{Flexural}\;\mathrm{strain}\;\varepsilon }_{f}=\frac{6\mathrm{Dd}}{L^{2}}$$

where F is the load at a given point on the load–deflection curve (N), L is the support span (mm), b is the width of the test beam (mm), d is the depth or thickness of the test beam (mm), and D is the maximum deflection of the center of the beam (mm). 

After the three-point bending tests, the fractured surface and the original unbroken surface were subjected to scanning electron microscopy (SEM; AURIGA, Zeiss, Oberkochen, Germany) in secondary electron mode and backscattered electron (BSE) mode at working distance 10 mm, accelerating voltage 15 kV to observe fracture patterns and microcrystalline structures.

### 2.4. Preparation of the Metal-Ceramic Specimens for Bond Strength Testing

**Figure 3:**
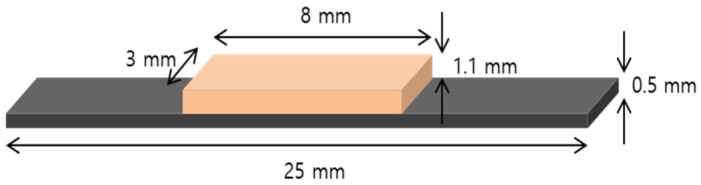
Schematic diagram of the shape of the Metal-Ceramic specimens. The orange part represents the veneering ceramic, and the black part represents the metal substrate.

In order to prepare the Metal-Ceramic bond strength testing, the metal parts of the Metal-Ceramic specimens were fabricated using the same devices and process (n = 12 for each group). The dimensions of the substrates were 25 mm × 3.0 mm × 0.5 mm, which is in accordance with ISO 9693-1:2012. After polishing, sandblasting, and cleaning, a layer of an opaque ceramic (Hera Ceram PO A2) was applied, and a body ceramic (Hera Ceram D A2) was fused to the central areas (8 × 3 × 1.1 mm) of the metal bars (Figure 3). According to the manufacturer’s instructions the firing procedure was carried out in a Programat p500 furnace (Ivoclar Vivadent, Schaan, Liechtenstein). Table 2 reports the firing schedules used for the ceramic.

### 2.5. Metal-Ceramic Bond Strength Testing

**Figure 4:**
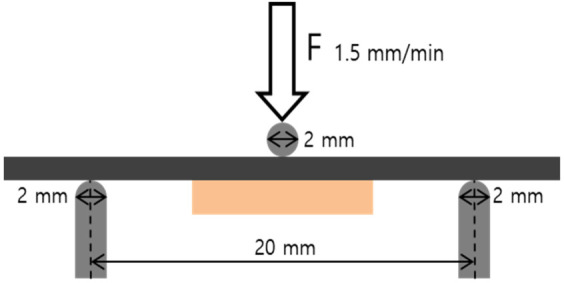
Schematic diagram illustrating the three-point bending test used to measure mechanical properties. The distance between the supports was 20 mm and the diameters of the bending piston and supports were each 2 mm. The bending piston was placed at the center and the crosshead speed of the loading part was 1.5 mm/min.

For Metal-Ceramic bond strength testing the Metal-Ceramic specimens were set up in a universal testing machine (TW-D102, Taewon Tech., Seoul, Korea). In accordance with ISO 9693-1:2012, the distance between the supports was 20 mm and the diameter of the bending piston was 2 mm. The specimen was placed at the center and the crosshead of the loading part was moved at 1.5 mm/min (Figure 4). The tests were run until debonding/cracking commenced. The bond strength (τ_b_) was calculated as follows:

$$\tau_{b}=F_{\mathrm{fail}}\left(A\times d_{m}^{2}+B\times d_{m}+C\right),$$

where A, B, and C are correction factors calculated using the elastic modulus of the three-point bending test and d_m_ is the thickness of the specimen (mm).

### 2.6. Fracture Mode Analysis

After Metal-Ceramic bond strength testing, fracture sites were examined for microstructure and fracture mode analysis. Five random specimens from each group were selected, and specimens were disassembled manually to analyze the fractured surface. The Metal-Ceramic interface was first observed by the naked eye, and images were taken using a digital camera (Nikon D5500, Nikon, Tokyo, Japan). Subsequently, the fractured surface was analyzed by means of SEM and energy dispersive X-ray spectroscopy (EDX) to analyze the distribution of Si remaining on the surface. EDX imaging was performed under working distance 15 mm, accelerating voltage 15 kV, and 170× magnification on an evaluation area of 1650 × 1200 µm at three different areas on each specimen. In addition, experimenter arbitrarily selected dark and bright spots on the surface enlarged by 1000× to analyze the components.

Failure modes were classified into three types: adhesive (less than 20% of the alloy surface covered by the remaining ceramic), mixed (20–80%), and cohesive (>80%) by means of software analysis of the Si detection data (ImageJ, NIH, Bethesda, MD, USA). The ratio of the area where the Si element was detected on the EDX image was calculated. This ratio was referred to as the Si remaining ratio.

### 2.7. Statistical Analysis

IBM SPSS Statistics Version 22 (IBM, Armonk, New York, NY, USA) was used for statistical analysis. Levene’s test was applied to assess the equality of variances. Data on roughness, elastic modulus, 0.2% yield strength, flexural strength, bond strength, and the ratio of Si remaining on the surface were analyzed by means of one-way analysis of variance (ANOVA) followed by Tukey’s test (α = 0.05). 

## 3. Results

### 3.1. Mechanical Properties testing

**Figure 5:**
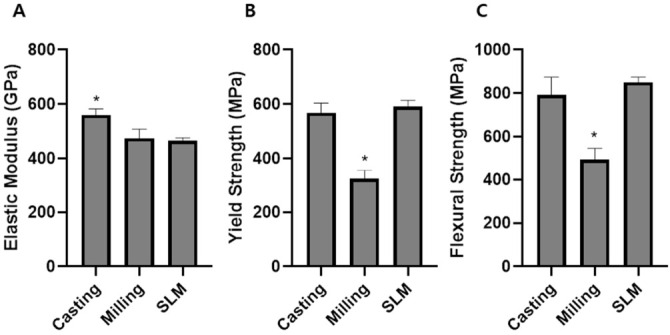
Mechanical properties of the casting, milling, and selective laser melting (SLM) groups: (**A**) elastic modulus, (**B**) yield strength, and (**C**) flexural strength. Asterisks (*) denote results statistically significantly different from those of the other groups (*p* < 0.05).

**Figure 6:**
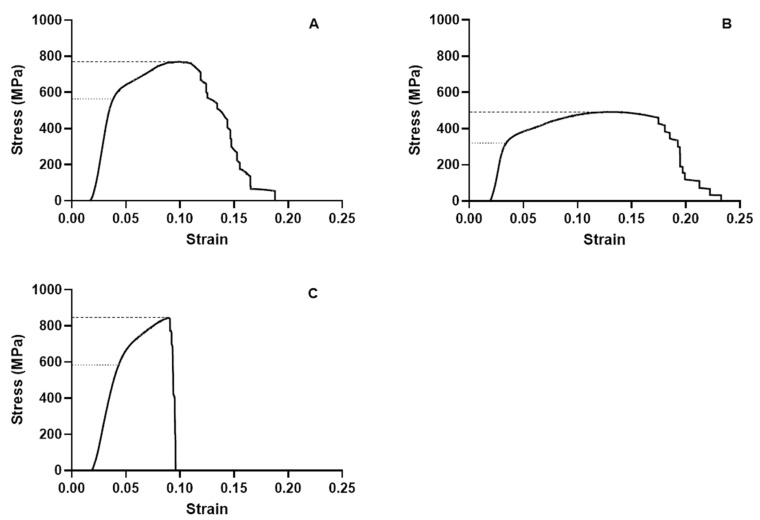
Mean stress–strain curves traced from the three-point bending test results: (**A**) casting, (**B**) milling, and (**C**) SLM. The dashed lines indicate the flexural strength, and the dotted lines indicate the yield strength. The elastic modulus can be calculated as the slope of the front linear part of the graph.

Table 3 and Figure 5 summarize the mechanical properties of the Co-Cr alloys of each group as calculated using measurements, whereas Figure 6 shows the stress–strain curve of each group. 

The elastic modulus was higher for the casting group (Figure 5A; *p* < 0.05). Differences in the yield and flexural strengths between the casting and SLM groups were not statistically significant, but these strengths were significantly lower for the milling group (Figure 5B,C; *p* < 0.05). The casting and SLM groups satisfied the minimum required elastic modulus values (150 GPa) and yield strength values (500 MPa) for class 5 of ISO 22674:2016, but the milling group showed lower yield strength (270–360 MPa).

### 3.2. Microstructure of the Alloy Surface

**Figure 7:**
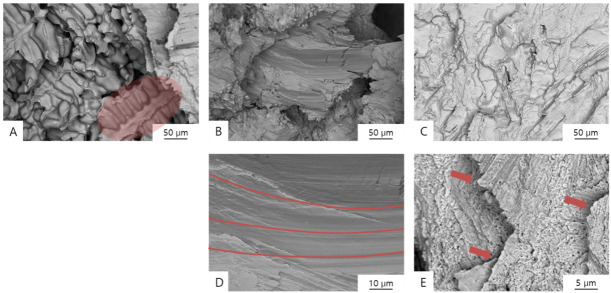
Backscattered electron (BSE) images of the fractured surfaces of Co-Cr alloy specimens formed by means of (**A**) casting (1000×; red shaded area indicates a dendritic structure), (**B**) milling (1000×), (**C**) SLM, (100×), (**D**) milling (5000×; red lines signify striations), and (**E**) SLM (10,000×; red arrows indicate cleavage steps).

**Figure 8:**
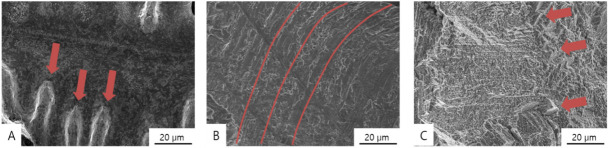
SEM images of the fractured surfaces of Co-Cr alloy specimens formed by means of (**A**) casting (4000×; red arrows indicate dendritic structures), (**B**) milling (4000×; red lines signify striations), and (**C**) SLM (4000×; red arrows indicate cleavage steps).

**Figure 9:**
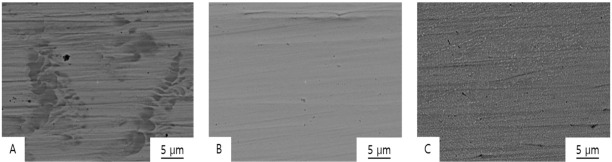
BSE images of the original unbroken surfaces of Co-Cr alloy specimens formed by means of (**A**) casting (10,000×; the dark shaded areas indicate dendritic crystal structures), (**B**) milling (10,000×; a uniform surface can be observed), and (**C**) SLM (10,000×; small crystalline structures that look similar to white grains can be observed).

Figure 7 and Figure 8 show SEM images of the fractured Co-Cr specimens, and Figure 9 shows the original unbroken smooth surfaces of the Co-Cr specimens.

Typical inhomogeneous dendritic and inter-dendritic solidification microstructures (Figure 7A, Figure 8A and Figure 9A) can found on the casting group, with grain sizes of approximately 50–100 µm. Contrastingly, large crystalline structures were not evident in the milling group specimens (Figure 9B), which showed homogeneous surfaces. In the SLM group, nanoscale crystalline structures were observed on the unbroken surface (Figure 9C), whereas a layered structure was observed at the fractured surface (Figure 7E and Figure 8C).

In the fractured surface of the casting group, it can be observed that the fracture occurred along the dendritic structure. On the fractured surface of a sample in the milling group, a wave pattern of striations was observed on the surface (Figure 7D and Figure 8B), which means that the specimen was stretched and fractured, implying that the material was ductile. Stair-like cleavage steps were evident on the fractured surface of the SLM group (Figure 7E and Figure 8C), which is usually seen with brittle materials.

### 3.3. Surface Roughness of the Metal Substrate

Table 4 lists the Ra values measured for each group. The casting group had the highest Ra, followed by the SLM and milling groups, but the differences between groups were not statistically significant (*p* > 0.05).

### 3.4. Metal-Ceramic Bond Strength of the Co-Cr Alloy

**Figure 10:**
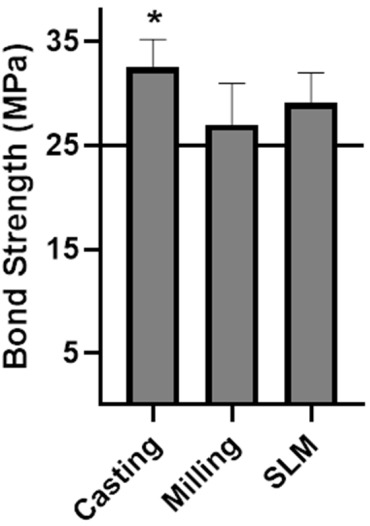
Metal-Ceramic bond strengths of the casting, milling, and SLM groups. The horizontal line at 25 MPa is the pass strength according to ISO 9693-1:2012. The asterisk (*) denotes results statistically significantly different compared with the other groups (*p* < 0.05).

Table 5 and Figure 10 present the results of the Metal-Ceramic bond strength experiments. The casting group showed the highest bond strengths (*p* < 0.05), whereas there was no significant difference between the other two groups. According to ISO 9393-1: 2012, at least four out of six specimens must have bond strength of at least 25 MPa to pass the test. Thus, all three groups passed the test.

### 3.5. Fracture Mode Analysis

**Figure 11:**
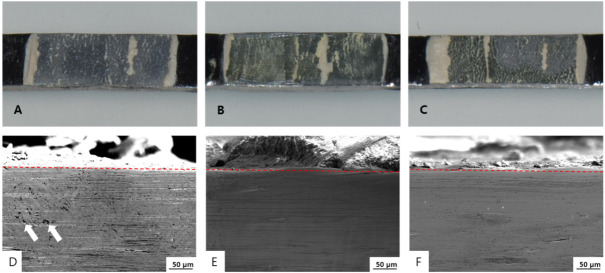
(**A**–**C**) Metal-Ceramic failure surface after separation of the ceramic layer: (**A**) casting, (**B**) milling, and (**C**) SLM specimens. (**D**–**F**) Cross-sectional SEM image of Metal-Ceramic specimens after debonding of the ceramic: (**D**) casting (the white arrows indicate defects), (**E**) milling, and (**F**) SLM specimens. Red dotted lines indicate the Metal-Ceramic boundaries.

All three groups showed irregular patterns of attached ceramic on the metal surface after failure (Figure 11A–C). After the three-point bending test, the ceramic was removed from the metal, and the metal surfaces were investigated with a digital camera and SEM. The lower part is the metal portion, and ceramic remains on the metal surface. In the casting group, small defects could be seen on the metal surface (Figure 11D–F).

**Figure 12:**
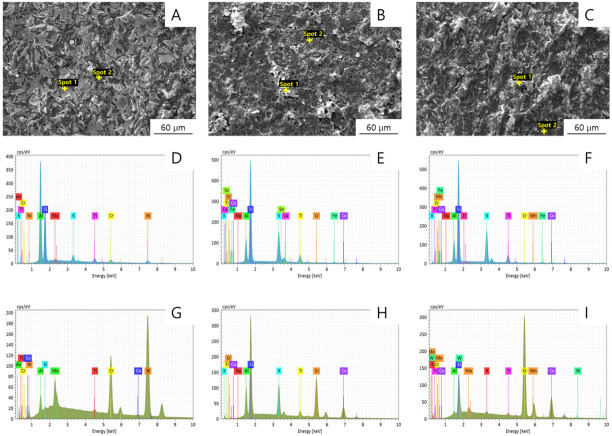
(**A**–**C**) SEM images (1000×) of (**A**) casting, (**B**) milling, and (**C**) SLM specimens. (**D**–**I**) EDX spectra of the Co-Cr alloy surface after removal of the ceramic layer: (**D**–**F**) spot 1 of the (**D**) casting, (**E**) milling, and (**F**) SLM specimens, and (**G**–**I**) spot 2 of the (**G**) casting, (**H**) milling, and (**I**) SLM specimens.

SEM and EDX analysis of the Co-Cr surface was conducted after manual separation of the ceramic layer (Figure 12). EDX analysis of the light spot (spot 1) and that of the dark spot (spot 2) shows different results. In all three groups, Si peaks can be found in spot 1. Compared with spot 1, a larger Cr peak can be found in spot 2. Therefore, it seems that the ceramic remains in the bright area and the metal surface is exposed in the dark area. After the three-point bending tests, the ratio of the area over which Si was detected was calculated by means of EDX mapping to determine the proportion of ceramic remaining on the surface. 

Table 6 lists the observed Si area ratios. A ratio of 20% or less indicates adhesive failure, whereas a ratio of 80% or more indicates cohesive failure. Ratios between 20% and 80% are regarded to indicate mixed failures. From the results of the analysis, the ratio increased in the following order: casting < SLM < milling. All specimens showed mixed failure patterns.

## 4. Discussion

In this study, the physical properties of the alloys and Metal-Ceramic bond strengths depended on their manufacturing method. Summing up the experimental results, the method of manufacturing the alloy does affect the properties of the metal.

Measuring the mechanical properties of alloys is very complex. Different standards and measurement methods are used to address the different needs of each field and situation [17,18,19,20]. For instance, the commonly used standard for measuring the mechanical properties of alloys used in dental prostheses is ISO 22674:2016 [21]. The casting group showed the highest elastic modulus; a higher elastic modulus means that more stress is required to deform a material by a given amount. The milling group showed the lowest yield strength and flexural strength. Lower yield strength means that plastic deformation occurs at lower stress levels, whereas lower flexural strength means that flexion fracturing occurs at lower stress levels. Zhou et al. [22] tested the mechanical properties of Co-Cr alloys fabricated by means of SLM, milling, and casting, and found that the SLM group showed higher yield strength, tensile strength, and elongation. Kim et al. [23] tested the mechanical properties of Co-Cr alloys fabricated by means of SLM, milling/post-sintering, milling, and casting, and reported that the milling group specimens were inferior to specimens of the other groups. Jabbari et al. [24] reported greater hardness of SLM specimens compared with casting specimens.

According to the stress–strain curves (Figure 7), the toughness and the total energy absorbed until fracturing was higher in the casting group. In the SLM group, fracturing occurred at a low strain level, so it can be considered the most brittle. Øilo et al. [25] also tested three-unit Co-Cr alloy bridges and showed that SLM framework specimens were brittle and harder than casting and milling specimens. The milling group showed the most deformation before fracturing, so it is relatively ductile, whereas the SLM group was relatively brittle due to fracturing under less deformation.

In the SLM manufacturing method, factors such as building direction, layer thickness, scan speed can affect the properties of the final product. In the present study, a 200 W Yb-fiber laser was used to build vertically, with a layer thickness of 20 µm and scan speed of 7 m/s. Takaichi et al. [26] reported that the yield strength in tensile tests is dependent on the sample’s building direction and that yield strength is higher for vertical printing compared with horizontal printing. Lu et al. [27] considered that the settings of speed 7 m/s, laser power 95 W, track width 0.11 mm, and layer thickness 25 µm are promising in terms of the resulting yield strength, corrosion resistance, and margin-fit accuracy.

Post-production heat treatment is also one of the factors that can affect the mechanical properties of metals. In this study, heat treatment was performed only in SLM among the three groups. Post-production heat treatment of SLM Co-Cr alloy is effective for releasing residual stress, thereby leading to a homogenized microstructure and improving mechanical properties [26,28]. Moreover, the tensile strength of Co-Cr alloy decreases slightly and the ductility increases slightly as the post-production heat treatment temperature and time increase [29]. Further study on post-production heat treatment in casting and milling groups is needed because the mechanical properties of the Co-Cr alloy may vary depending on the heat treatment conditions.

According to the results of the present study, the mechanical properties of alloys varied depending on the manufacturing method [30], with the casting and SLM group specimens showing values better than those required by the ISO 22674:2016 standard. Although the yield strength and flexural strength of the milling group were the lowest, this material was still suitable for use in class 3 applications (multiple-unit fixed prostheses).

The casting group showed the highest Metal-Ceramic bond strengths, meaning that it was more difficult to separate the ceramic and metal parts. The reason for the differences in bond strength among groups can be explained by their differences in elastic modulus, chemical bonding, mechanical interlocking, and compressive bonding. When comparing the Metal-Ceramic bond strengths of alloys produced by means of SLM and casting, Xiang et al. [31] and Wang et al. [32] reported higher bond strengths in the SLM group. On the other hand, Kaleli et al. [33], Li et al. [34], and Wu et al. [35] reported that there were no statistically significant differences in bond strength between the casting, milling, and SLM groups. In addition, another study [36] reported that a milling group showed high Metal-Ceramic bond strength, whereas a casting group showed low bond strength. 

In general, alloys with higher moduli of elasticity are more resistant to bending and peeling, resulting in stronger Metal-Ceramic bonding as seen in the formula for obtaining bond strength,

$$\tau_{b}=F_{\mathrm{fail}}\left(A\times d_{m}^{2}+B\times d_{m}+C\right)$$

Because the bond strength is obtained through the failure force (F_fail_) and constants A, B, and C, it is likely that the high elastic modulus could have caused the high bond strength seen in the casting group. In addition, ceramics are brittle and vulnerable to deformation, and when a ceramic is combined with a metal having low elastic modulus, ceramic fracturing can easily occur due to the facile elastic deformation of the metal, even under low forces.

Chemical bonding occurred by means of chemisorption due to diffusion at the Metal-Ceramic interface and was affected by the oxide layer at interface [37]. Xin et al. [38] found that a thicker oxide layer was formed on the surface of the Co-Cr alloy produced by the SLM method than that produced by casting, which can affect the chemical bonding at the Metal-Ceramic interface. How the thickness of the oxide layer varies depending on the method of manufacturing the metal and how it affects the Metal-Ceramic bond strength is an area requiring further study.

Mechanical interlocking is another factor influencing bond strength. In general, high roughness is known to help increase Metal-Ceramic bond strength [39,40]. It was found in this study that the Ra values of the Co-Cr alloy surfaces measured were not significantly different among groups. However, this may not be the case when the surface properties are the same but the standard deviation is large. In this study, only simple rubber polishing was performed without high-polishing so as not to apply excessive heat to the surface or change the surface properties. Therefore, it seems that the standard deviation of the surface roughness appeared large. The difference between the three groups was not significant, further research is required to determine the effects of the alloy surface.

Compressive bonding is also one of the factors affecting Metal-Ceramic bond strength. When the coefficient of thermal expansion (CTE) value of the metal is slightly higher than that of the ceramic, this is referred to a positive mismatch. This causes the formation of strong Metal-Ceramic bonds during the cooling process after ceramic firing. In the present study, the CTE of the casting ingot was higher than that of the ceramic, whereas the materials used for milling and SLM had similar CTEs as the ceramic. This difference would have contributed to the higher bond strength observed for the casting group.

Only the SLM method included post-production heat treatment in this study. According to Yan et al. [41], there was no difference in bond strength between SLM Co-Cr alloys treated at 880 or 1100 °C, whereas Xin et al. [42] reported that the ceramic firing process did not change the surface structure of the SLM alloy.

There is a method for treating the metal surface to increase Metal-Ceramic bond strength. Dimitriadis et al. [43] reported a slight decrease in bond strength when a bonding agent was used on the surface of an SLM Co-Cr alloy. Furthermore, Al Bakkar et al. [44] reported that the bonding agent on the Co-Cr surface had a minor effect on bond strength. Sandblasting of the alloy surface can also affect bond strength. Park et al. [45] reported that acid etching and sandblasting of the surface of Co-Cr alloy helps to increase bond strength, whereas Külünk et al. [46] reported that sandblasting with 110-μm Al_2_O_3_ was better than that with 50-μm Al_2_O_3_ as a means to increase bond strength. In this study, samples were sandblasted with 80-μm Al_2_O_3_ particles and no bonding agent was used.

Furthermore, marginal fit of the prosthesis is also an important factor in clinical practice. Nesse et al. [47] reported that three-unit bridges showed good marginal fit in order of milling, casting, and SLM. Akçin et al. [48] also reported that casting showed the best marginal fit followed by SLM, and milling showed poor fitness in a five-unit bridge. However, Dicova et al. [49] reported experimental results that SLM has better marginal fit than casting in a three-unit bridge. If the marginal fit of SLM is improved, it will have more clinical advantages.

To summarize the results of this study, the SLM method satisfied the ISO 22674:2016 and ISO 9693-1:2012 standards for manufacturing dental prostheses. SLM can be considered a suitable method for fabricating Co-Cr alloys for clinical use because the resulting product exhibits mechanical properties and Metal-Ceramic bond strength values beyond the requirements, and because this manufacturing method has several advantages including good productivity and reduced human error.

## 5. Conclusions

Within the limitations of this study, the following conclusions were drawn. The mechanical properties of Co-Cr alloy depend on the manufacturing method. Specimens in the casting group showed high elastic moduli, whereas those in the milling group showed low yield strengths and flexural strengths. All three groups exceeded the ISO standards for Metal-Ceramic bond strength. The SLM technique can be used for manufacturing dental prostheses according to ISO 22674:2016 and ISO 9693-1:2012. Considering its many other advantages, the SLM method seems to have the potential to replace traditional fabrication methods.

## Figures and Tables

**Table 1:** Specifications of the materials used in this study.

Group	Brand Name	Composition (wt%)	CTE (×10^−6^ K^−1^)	Manufacturer
Casting	Star Loy C	Co 59.4%, Cr 24.5%, W 10%, Nb 2%, V 2%, Other (Mo, Si, Fe) ≤ 1%	14.6–14.9	Dentsply Sirona, York, PA, USA
Milling	Starbond Co-Cr block	Co 59%, Cr 25%, W 9.5%, Mo 3.5%, Other (Si, C, Fe, Mn, N) ≤ 1%	13.9–14.2	Scheftner dental alloys, Mainz, Germany
SLM	SP2	Co 62%, Cr 24%, Mo 5%, W 4%, Other (Si, Mn, Fe) ≤ 2%	13.9–14.3	EOS, Krailling, Germany
Ceramic	Hera Ceram	Glass-based ceramic	13.5–14.9	Heraeus, Hanau, Germany

Information provided by the manufacturers. CTE: coefficient of thermal expansion.

**Table 2:** Firing schedules used in the ceramic veneering procedure.

Product Name	Pre-Heating Temp. (°C)	Drying Time (min)	Heating Rate (°C/min)	Final Temp. (°C)	Holding Time (s)	Vacuum
Degassing	500	1	50	980	50	+
First opaque	500	10	50	960	50	+
Second opaque	500	10	50	950	50	+
Dentin	500	5	50	930	38	+
Glaze	500	2	50	901	38	+

Note: +, The firing was done under vacuum.

**Table 3:** Mechanical properties of the specimens.

Group	Elastic Modulus (GPa)	0.2% Yield Strength (MPa)	Flexural Strength (MPa)
Casting	560.53 ± 21.53 *	567.92 ± 35.53	792.31 ± 81.64
Milling	473.55 ± 35.02	323.86 ± 32.04 *	494.16 ± 51.93 *
SLM	464.55 ± 10.77	591.18 ± 22.31	849.48 ± 24.45

Results are expressed as means ± standard deviations. Asterisks (*) denote results statistically significantly different compared with the other groups (*p* < 0.05).

**Table 4:** Surface roughness of the specimens.

Group	Ra (µm)
Casting	1.19 ± 0.58
Milling	0.88 ± 0.46
SLM	1.10 ± 0.30

Results are expressed as means ± standard deviations. Ra: profile surface roughness.

**Table 5:** Metal-Ceramic bond strength and passing rate (≥25 MPa) of the specimens.

Group	Bond Strength (MPa)	Passing Rate
Casting	32.51 ± 2.68 *	100.00%
Milling	26.98 ± 3.97	66.67%
SLM	29.07 ± 2.90	91.67%

Results are expressed as means ± standard deviations. Asterisks (*) denote results statistically significantly different compared with the other groups (*p* < 0.05).

**Table 6:** Failure mode analysis results and the area fraction of Si detected.

Group	Area Fraction of Si Detected (%)	Failure Mode
Casting	36.44 ± 6.37 ^a^	Mixed
Milling	57.10 ± 12.26 ^b^	Mixed
SLM	49.50 ± 7.69 ^a,b^	Mixed

Results are expressed as means ± standard deviations. Different lowercase letters (^a^, ^b^, and ^a,b^ in the table) indicate significant differences between groups (*p* < 0.05).
